# Sensorized Skin With Biomimetic Tactility Features Based on Artificial Cross‐Talk of Bimodal Resistive Sensory Inputs

**DOI:** 10.1002/advs.202301590

**Published:** 2023-09-07

**Authors:** Antonia Georgopoulou, David Hardman, Thomas George Thuruthel, Fumiya Iida, Frank Clemens

**Affiliations:** ^1^ Department of Functional Materials Empa ‐ Swiss Federal Laboratories for Materials Science and Technology 8600 Switzerland; ^2^ Bio‐Inspired Robotics Lab Department of Engineering University of Cambridge CB2 1PZ UK; ^3^ Department of Computer Science University College London E20 2AF UK

**Keywords:** deep learning, multimodal perception, soft sensors

## Abstract

Tactility in biological organisms is a faculty that relies on a variety of specialized receptors. The bimodal sensorized skin, featured in this study, combines soft resistive composites that attribute the skin with mechano‐ and thermoreceptive capabilities. Mimicking the position of the different natural receptors in different depths of the skin layers, a multi‐layer arrangement of the soft resistive composites is achieved. However, the magnitude of the signal response and the localization ability of the stimulus change with lighter presses of the bimodal skin. Hence, a learning‐based approach is employed that can help achieve predictions about the stimulus using 4500 probes. Similar to the cognitive functions in the human brain, the cross‐talk of sensory information between the two types of sensory information allows the learning architecture to make more accurate predictions of localization, depth, and temperature of the stimulus contiguously. Localization accuracies of 1.8 mm, depth errors of 0.22 mm, and temperature errors of 8.2 °C using 8 mechanoreceptive and 8 thermoreceptive sensing elements are achieved for the smaller inter‐element distances. Combining the bimodal sensing multilayer skins with the neural network learning approach brings the artificial tactile interface one step closer to imitating the sensory capabilities of biological skin.

## Graphical abstract

Inspired by the sensory characteristics of the human skin, a multi‐layer artificial skin with thermoreceptive and mechanoreceptive sensing elements is featured. Neural network processing allows the recognition and localization of the tactile stimulus over an area of the skin. The tunable architecture suggests the potential to adapt to dynamically changing environments for implementations in real‐life applications.

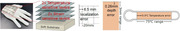

## 1 Introduction

The skin in a natural organism is typically the largest sensory organ, as it involves multiple specialized neurons that convey important sensory information to the brain about the surroundings.^[^ ^1^, ^2^ ^]^ Electronic skin attempts to mimic the sensory capabilities of natural skin, primarily tactility, using artificial sensors.^[^ ^3^, ^4^ ^]^ While there has been significant progress in the development of stretchable electronics that can enable the detection of tactile stimuli, the applicability of such devices remains limited.^[^ ^5^, ^6^ ^]^ This is primarily because of the challenges associated with developing fully stretchable electronic skin without suffering from material non‐linearities like hysteresis and drift.^[^ ^4^, ^7^ ^]^ These challenges are amplified when multi‐modal sensing functionalities are added. Recent review articles agree that there is a great need for soft functional materials with multi‐modal sensing capabilities and more precise data processing algorithms to improve the applicability of e‐skin.^[^ ^7^, ^8^, ^9^, ^10^ ^]^ There are sensory features that can be adopted from nature and integrated into sensorized e‐skin.

**Figure 1:**
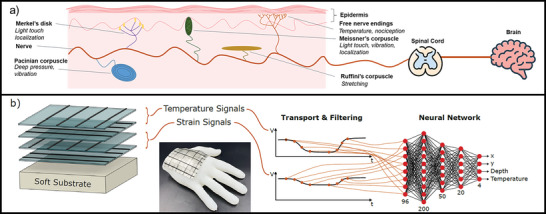
a) Schematic of the modalities of mechano‐ and thermoreception found in the human skin. b) The biomimetic sensorized skin and processing methods presented in this work.

Having e‐skin that closer resembles the tactile capabilities of natural skin can improve the performance and control of robots, human/machine interfaces, and prosthetic devices. Natural skin possesses specialized sensory receptors for conveying dedicated information (**Figure** **1**a). In the epidermis, free nerve endings can detect changes in the temperature, attributing the capability of thermoreception.^[^ ^11^, ^12^ ^]^ Specialized mechanoreceptors can be found in the encapsulated form. Merkel's discs in the dermis and epidermis are responsible for the sensation of light touch.^[^ ^13^ ^]^ Meissner's corpuscles are found beneath the epidermis and detect low frequency vibrations and light touch.^[^ ^14^, ^15^ ^]^ Both Merkel's discs and Meissner's corpuscles are finely calibrated and can precisely localize tactile stimuli.^[^ ^16^ ^]^ Pacinian corpuscles and the Ruffini endings reside deeper in the dermis and subcutaneous tissue.^[^ ^17^ ^]^ They are responsible for detecting pressure/high frequency variation and cutaneous stretching, respectively.^[^ ^18^ ^]^ These two mechanoreceptors respond to deep pressure, but cannot detect the fine localization of the tactile stimulus.^[^ ^19^, ^20^ ^]^ The specialization of different receptors found in the skin and their arrangement in different layers can inspire the placement of the artificial receptors in multi‐layer formation, as will be seen in this study.

The processing of sensory information in biological organisms can also inspire the development of e‐skin. While sensory receptors are responsible for detecting the presence of a tactile stimulus, the brain encodes the message and transforms it into relevant information for perception and action. An interplay of the signal of the different mechanoreceptor types is transmitted to the somatosensory cortex of the brain and can be processed to relevant information about the time, location, temperature, and intensity of the tactile stimulus (Figure 1a).^[^ ^21^, ^22^, ^23^ ^]^

The function of perception involves the interpretation of a sensation in the brain.^[^ ^24^ ^]^ Millions of sensory neurons constantly transmit information that the human brain can identify, organize, and interpret sensory information with the perception process.^[^ ^25^ ^]^ For the learning process, the brain must streamline its data processing and tune its sensitivity to relevant processes.^[^ ^26^, ^27^, ^28^, ^29^ ^]^ Although the exact neural circuitry of touch and thermal perception is not well understood, observations suggest that there is some level of cross‐talk among different somatosensory modalities.^[^ ^30^, ^31^ ^]^ This type of cross‐talk of bimodal sensory information can be used as a source of inspiration for the e‐skin and neural network processing (Figure 1b).

The development of e‐skin with the ability to detect multiple stimuli is an essential problem in the development of prostheses and robotic systems.^[^ ^32^, ^33^, ^34^ ^]^ Multifunctional stretchable sensory skins for detecting pressure, proximity, temperature, etc. have recently been developed by stacking planar sensing layers with different functionalities^[^ ^35^, ^36^ ^]^ or by using 3D structures that are sensitive to physical stimuli.^[^ ^37^, ^38^ ^]^ Nonetheless, most of these technologies still incorporate rigid components in their design making them non‐stretchable and their sensing region is typically discrete. Another major challenge is the perception of multiple stimuli at the same time due to mutual interference.^[^ ^3^, ^39^ ^]^ Typically, this cross‐coupling effect is reduced to achieve multi‐modal sensitivity.^[^ ^39^, ^40^ ^]^ Tactile sensing is often used in wearable electronic devices.^[^ ^41^, ^42^ ^]^ Flexible sensors based on polymeric materials and composites can be used for detecting contact, while maintaining softness and stretchability, like biological skin. This type of sensing can be used for detecting when contact with an object has occurred^[^ ^43^, ^44^, ^45^ ^]^ and typically the applied pressure can be quantified.^[^ ^46^, ^47^ ^]^ Recently, sensing that can relay simultaneous information about the applied force, strain, and temperature has become available.^[^ ^48^, ^49^ ^]^ However, achieving selectivity to one stimulus and at the same time obtaining information about the presence of multiple stimuli remains a challenge.^[^ ^50^ ^]^ Looking from an information theory viewpoint, however, cross‐talk and mutual interference are not necessarily detrimental and in some cases can be advantages for state estimation. Such interelement interactions can be used for improving robustness to damage,^[^ ^51^ ^]^ reducing modeling errors^[^ ^52^ ^]^ or compensating for external environmental changes.^[^ ^53^ ^]^ Typically, learning‐based approaches are used in such cases, similar to the methodology in this paper.^[^ ^4^, ^37^, ^54^, ^55^ ^]^

In this work, biomimetic tactility will be investigated in a sensorized e‐skin produced with material extrusion based additive manufacturing (MEX‐AM). Similar to the free nerve endings closer to the skin surface for temperature detection, two layers with integrated flexible thermistors were placed on top. Underneath, two layers with piezoresistive sensing elements, resembling the function of the Pacinian and Ruffini's corpuscles, were included. Their function is to detect the presence of pressure due to the deformation of the skin (piezoresistive response). For localizing the stimulus and measuring its magnitude, which is the function of Meissner's corpuscles and Merkel's discs, the piezoresistive sensing elements were used in combination with a learning‐based algorithm. Further details are presented in Section 4. It was assumed that the entire skin is flexible and stretchable, making it easy to integrate into existing robots. Unlike related works on multi‐modal tactile sensing,^[^ ^39^, ^40^, ^56^, ^57^ ^]^ in the current study, the idea is to sense light touch, depth, and temperature contiguously. Inspired by the cross‐talk of multi‐modal sensory information in the brain, it is demonstrated how the fusion of information from the temperature and deformation receptors lead to better perception capabilities for both modalities. Such characteristics will pave the path for the foundation of artificial cognitive perception, a function that will enhance the applicability of stretchable electronics and affect several fields including soft robots, wearable, and prosthetic devices.

## 2 Results and Discussion

### 2.1 Characterizing the Sensory Receptors: Single Point Measurements

**Figure 2:**
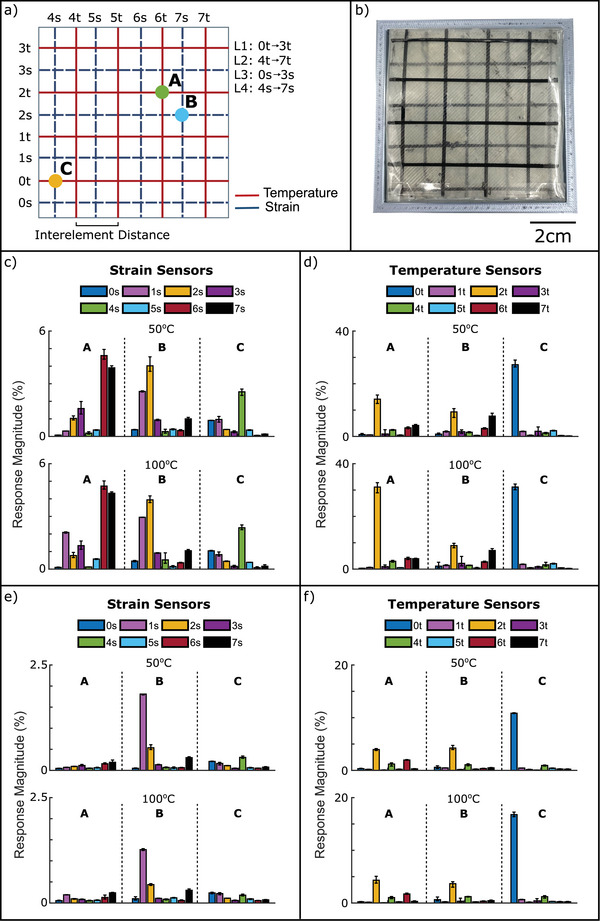
a) Schematic representation of the mechanoreceptive and thermoreceptive sensing elements arrangement in the sensorized skin and the definition of the three probed points during the testing. b) Photograph of the bimodal sensorized skin. Response at 50 and 100 °C for probing depth of 4 mm (deep pressure) for c) the mechanoreceptive and d) the thermoreceptive sensing elements, over four repetitions. Response at 50 and 100 °C for probing depth of 1 mm (light touch) for e) the mechanoreceptive and f) the thermoreceptive sensing elements, over four repetitions.

The response of the sensing elements was characterized during the application of mechanical or thermal stimuli with the probe of the robot at the defined point (**Figure** **2**a,b). The two sensor materials have been optimized for having good sensitivity to a specific stimulus. The thermoresistive sensor has a high thermal expansion coefficient base on semi‐crystalline structure. A low carbon filler has been selected below the percolation threshold to achieve high change in electrical resistance by small change in volume expansion.^[^ ^58^ ^]^ In contrast, to achieve high sensitive mechanoresistive sensor properties, a high filler content is favored to achieve monotonic increase of resistivity in a large strain area. The increase in stain will result in an increase of the interparticle distance of the carbon black leading to the positive piezoresistive response. To avoid thermal resistive effect, an amorphous polymer matrix has to be selected.^[^ ^58^ ^]^ With an increase in the strain as a result of the skin stretching, the distance of the particles increases.

First, the deep pressure test took place with a probing depth of 4 mm (Figure 2c,d, with the time series responses given in Figure S1, Supporting Information). The response of the mechanoreceptive elements at point A was low, and the elements 2s, 3s, 6s, and 7s (surrounding point A) resulted in a low relative signal change of 1%, 1.4%, 4.5%, and 4%, respectively. This can be expected because point A is the crossing point of two thermoreceptive elements. The elements 6s and 7s on L4 (the fourth layer from the top), showed a two to three times higher relative signal change than the 2s and 3s elements on L3. By applying a probing depth of 4 mm, the soft e‐skin structure was deformed under a bending mode. Therefore, the lower the layer from the top, the higher the strain deformation. A higher deformation resulted in a higher resistance change of the mechanoreceptor element and therefore, it was expected that the sensors on the fourth layer would result in a higher relative signal change. It is worthwhile to mention that no significant resistance change could be observed for all other mechanoreceptive elements. According to this result, it was assumed that the e‐skin was only deformed locally. The response of the mechanoreceptive elements did not change more than 0.2% after the heating. This shows that the mechanoreceptive sensors are not significantly affected by the local heating of the elastic e‐skin and thus, they exhibit selective response to mechanical stimuli.

Looking at the thermal response of the sensing elements when point A was probed, it can be seen that there was a change in the signal response for the 2t and 6t elements (Figure 2d). Element 2t produced a response of magnitude of 15% and element 6t of 4%. Element 2t was on L1 (top layer) and element 6t in L2 (second layer). The differences in the magnitude of the response can be explained by the lower conductivity of the substrate L1 that is in‐between the 2t and 6t crossing point. Unexpectedly, there was also a change in the response of the elements 4t (3.5%) and 7t (4%), proximal to point A and this was attributed to the stretching of the skin. However, this was not seen for the other elements (1t and 3t on L1 and 5t on L2). A temperature increase to 100°C resulted in a higher relative signal change by the thermoreceptive element 2t (31%). For the 4t, 6t, and 7t elements, the response remained the same. Only the thermoreceptor 2t was in direct contact with the probe. As mentioned before, the low thermal conductivity of the support material significantly affected the performance of the thermoreceptors if they were not in direct contact with the heated probe. Longer term temperature responses over 10 min can be seen in Figure S2 (Supporting Information).

Point B is the crossing point of mechanoreceptive elements 2 and 7s (Figure 2c). Here, the 2s resulted in a relative signal response of 4.2%, whereas the relative signal response of the 7s was only 1%. A relative signal change of 2.3% and 1% for the elements 1 and 3s shows, that in this case, the deformation of the e‐skin is more lateral due to the softer support material closer to L1 and L2. This assumption correlates with the 6s sensor signal, which did not significantly change, and is placed in the fourth layer. The relative signal change was similar when the probe was heated to 100°C. This is in good agreement with the results discussed for point A and therefore the mechanoreceptive sensors are not sensitive to temperature changes.

For the thermoreceptive elements near point B, a relative signal change of 8.5% (2t), 2% (6t), and 7.5% (7t) could be observed. All these three elements were proximal to point B. Unfortunately, the relative signal was similar when increasing the probe temperature up to 100°C. The low thermal conductivity of the support material did not transport the heat into the proximal thermoreceptive elements.

Point C was the crossing point of elements 0t and 4s. For deep pressure (4 mm depth) and a temperature of 50°C, the thermoreceptor element 0t resulted in a relative signal change of 26% and the mechanoreceptor 4s in a change of 2.4% (Figure 2e). This was in good agreement with the results achieved at point A, even though the relative resistance change for both receptor elements was different. It was assumed that the difference in relative signal change between points A and C was caused by the fact that the probing was proximal to the frame, where the elastic e‐skin was fixed. However, when the temperature of the probe increased to 100° the mechanoreceptor signal change did not vary significantly, whereas the signal response of the thermoreceptive elements increased, as expected.

For the light touch test (probing 1 mm depth) the sensor response was examined at the same points (Figure 2e, f) with time series responses given in Figure S3, Supporting Information). For point A, all mechanoreceptive elements resulted in a very small signal change (<0.1%) that was not affected by the temperature change. The selectivity of the sensor response was in good agreement with the results of the deep pressure test. Selective sensors exhibit high sensitivity to one stimulus and minimal response to other interfering stimuli.^[^ ^50^ ^]^ The thermoreceptive elements 2t and 6t produced relative signal changes of 4% and 1.8%, respectively. This response was significantly smaller than the one seen during the deep pressure test. When the temperature increased to 100 °C, only 2t element showed a significant increase of the relative signal (4.5%), as expected. It was evident that the thermoreceptive elements were not selective to the temperature stimulus.

For point B, mechanoreceptive element 1s resulted in a signal change of 1.5% and this did not significantly change by increasing the temperature. As expected, this value was significantly smaller in comparison to the deep pressure tests (4 mm). For element 2t, a relative signal change of 4% and 4.3% for the two different temperatures 50 and 100°C was observed, respectively. Similar to point A, the temperature signal differed when compared to the deep pressure test. For the point C, all mechanoreceptive elements resulted in a very small signal change (< 0.1%), independent of the probe temperature. Only the thermoreceptive element 0t resulted in a relative signal change of 11%. Similar to points A and B, this relative change was lower in comparison to the deep pressure test. By increasing the temperature of the probe to 100%, the relative signal change increased to 17%.

Based on the single point measurements, it was concluded that the mechanoreceptor elements were not significantly affected by the temperature window (50 and 100°C), whereas the signal of the thermoreceptor elements was affected by the probing depth (deep pressure and light touch tests). During the light touch tests (1 mm), the mechanoreceptive elements did not detect the location of the tactile stimulus. The neural network learning approach was necessary for being able to analyze experiments with low probing depth. It is worthwhile to mention that due to the short probing time (10 s), it was not possible to transfer the heat through the TPU support material, due to its low thermal conductivity.

**Figure 3:**
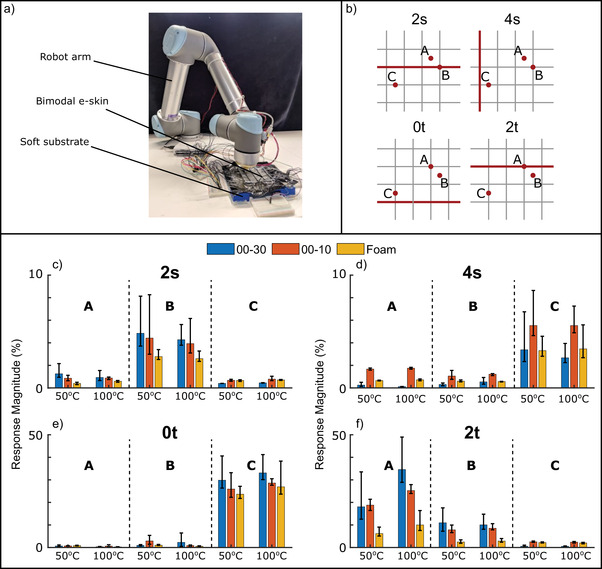
a) Photograph of the robot arm performing the probing. b) Probed locations with respect to the tested sensors. c–f) Differences in response magnitude of four selected sensors to a variety of 4 mm deep presses, when one of the four different substrates (Ecoflex 00–30, Ecoflex 00–10, and silicone foam) are placed underneath the large skin for sensing elements c) 2s d) 4s e) 0t f) 2t. five repetitions are performed for each. The percentage deviation was calculated from the baseline resistance during each response. The red line indicates the sensor element that was being investigated in each subfigure.

To investigate the effect of the stiffness of soft tissue, two Ecoflex materials and one silicone foam were placed underneath the e‐skin. Based on previous results, the deep pressure test was used to investigate the stiffness effect on the sensitivity of the receptor elements. Two mechanoreceptive and two thermoreceptive sensory elements were examined for the selected locations (A, B, and C) at 50 and 100 °C (**Figure** **3**a,b). It was seen that that the response was more sensitive when probing location B, exhibiting a localization ability (Figure 3c). The differences between the three different substrates were not significant, but there is a slightly smaller sensitivity for the silicone foam material. Since point B is at the intersection of two mechanoreceptive elements (2s and 7s), there was not a significant dependency upon the temperature change. In the case of element 4s (Figure 3d), localization ability was seen when point C was probed. However, in this case, the 00–10 substrate (lowest Shore hardness) gave the highest response magnitude. This difference was attributed to the deformation of the skin not transporting to the lowest layer (L4) evenly for all substrates, resulting in an asymmetric response. A similar asymmetry was observed for the mechanoreceptive elements 0t and 2t (Figure 3e, f), but in this case, the low heat transfer was considered to be the cause.

### 2.2 Tactile Stimulus Predictions

**Figure 4:**
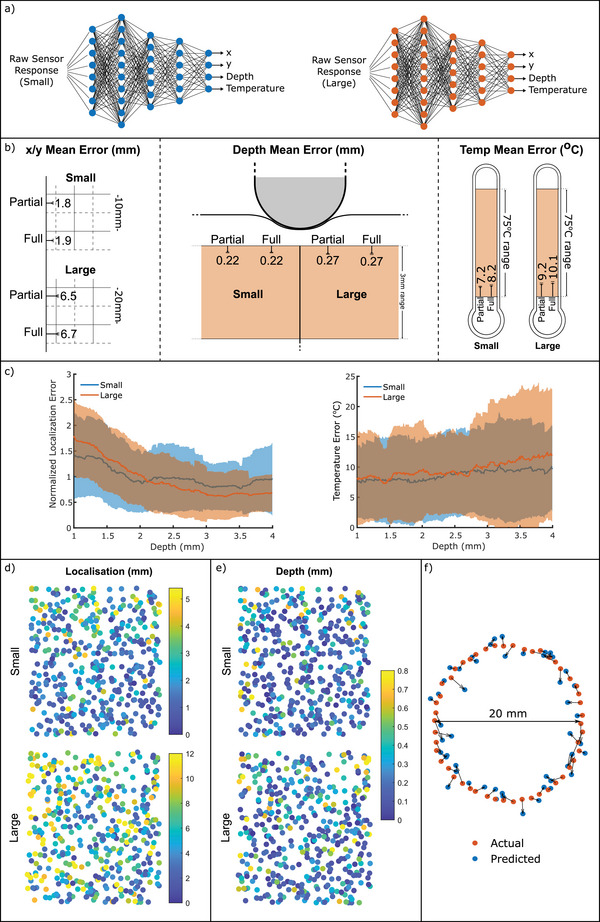
a) Using a trained network: raw responses are fed in to a network for the size under consideration, and four predictions are output. b) Mean test set (*n* = 500) prediction errors of the trained networks, repeated five times with changes in the output targets. c) Dependence of prediction errors on the depth of pressing. Localization errors are scaled to have a mean of one for comparison purposes d) x‐y localization error distributions in the test sets (*n* = 500), for networks trained separately on the large and small skins. The color bars are scaled proportionally with the skin size. e) Depth error distribution of the same networks. f) Actual location of probing (orange) versus predicted location of probing (blue) for a circle traced on the small skin.

Section 2.1 has demonstrated the initial feasibility of the e‐skin, mechanoreceptive elements responded selectively to strain, whereas the thermoreceptive elements responded to temperature and strain. In addition, it was demonstrated that the response of the sensors depended on the probing depth and the proximity of the sensing element to the stimulus. Inspired by the biomimetic skin structure, temperature sensors placed uppermost (Figure 1) were found to be the most sensitive, detecting changes in temperature as well as local strains. In real‐life applications, the usefulness of a sensor is not only given by its response signal but also by how well its response can be interpreted and aspects, like the tactile stimulus recognition (temperature or pressure) and localization are essential. Thus, the primary purpose of the featured sensors lies in their capability to recognize, locate and categorize tactile sensory inputs. To that end the raw data analysis from Figures 2 and 3 was used as input for the neural network, which was trained to output three parameters of the stimulus (lateral location, depth, temperature), as described in Section 4. **Figure** **4**a illustrates the expected workflow once the networks have been trained: raw responses were directly mapped to the predictions, with separate networks for small and large skin sizes. This analysis is a useful process for handling large sets of data and different types of sensory information to achieve localization and recognition of the sensory stimulus with good accuracy.

The mean prediction errors of neural networks trained using both the mechanoreceptive and thermoreceptive sensor responses are presented in Figure 4b. Each of the sensory outputs i.e., localization, depth, or temperature was trained separately by Partial Output. Conversely, Full Output predicted all values simultaneously. With no changes in architecture, switching between the two had a negligible effect on the prediction errors and since the Full Output model was more compact and data‐efficient, the networks were trained using a Full Output for all subsequent figures.

Using this Full Output, the small skin's network was able to localize the test set's presses with a mean error of 1.8 mm. This value was considerably less than the resolutions achievable by looking at the highest sensor responses without the network (8.9 mm between adjacent strain sensors in the grid, or the 4.4 mm between sensors of any type). Similarly, the large skin's network localized with a sub‐grid resolution, with a mean error of 6.6 mm compared to the 17.8 mm between adjacent strain sensors. Though the size was doubled, the localization error increased by 270%, due to the fact that the probe diameter and the depth of pressing did not scale accordingly. Changing these parameters would be expected to result in a shift in mean error, and could quickly be accounted for by retraining just the network's final layer.^[^ ^59^ ^]^

In Section 2.1, it was hypothesized that a network's localization performance would improve with the probing depth. Thus, Figure 4c fulfilled these expectations for both the small and large skin. As seen in Figure 2, the mechanoreceptive sensor layers localized light presses less accurately than deeper presses, especially within the first few mm of pressing. This observation agreed with the findings of analysing the raw sensor data response. Even with the neural network processing, for the small skin, 2 mm of depth was required for the error to reach a constant value, while this value is closer to 3 mm for the large skin. Designing a biomimetic skin to operate within this stable region is a sensible way of ensuring high localization performance, depending on a number of layers, layer thickness and substrate selection. There is a limitation in the depth of placing the sensory elements that significantly affects the localization precision.

As for the depth prediction errors, the difference between small and large sizes was smaller than the localization predictions, as observed in Figure 4b. Since the range of probing depths did not change between experiments, the small skin's network gave a mean error of 0.22 mm, increasing to 0.27 mm in the large skin. Similarly, the error in temperature predictions was 8.2 °C (small skin) and 10.1 °C (large skin) of the ≈70 °C range. The networks significantly outperformed a naive prediction of the average value, which would give mean errors of 16.0 and 17.45 °C. Unlike the x/y localization, there was no dependency on the probing depth in the error of predicted temperature, as illustrated in Figure 4c. The large skin's average error begins to increase at the deepest stimuli, which was attributed to the increased likelihood of the deeper presses producing noise in the connections. In Figure 2, it was seen that the response of the mechanoreceptive elements on deformation depended on the depth of the layer and was highly influenced by asymmetry effects. Using neural network processing with the cross‐talk between the two type of sensing elements, the x/y location and depth of the deformation by press could be localized more precisely, regardless of the location of the probing in the studied area.

Figure 4d shows the distributions of the test set localization errors for the two skin sizes, with the color bars scaled for equivalence. As before, it was seen that the localization error of the large skin was larger than the skin's dimensions, but also that many of the larger errors were clustered on the left side of the grid. Therefore, for the large skin, the prediction failed to localize the stimulus, particularly for probings close to the left side of the grid. A possible justification for this effect could be a loose connection leading to the asymmetrical error distribution. The same effect was not apparent in the small skin, where the nearby strain sensor was at the same four‐layer depth. In that case, larger errors appeared disproportionately in the upper half of the characterization area. A similar asymmetry in the response was also observed for the raw data analysis and was associated with limitations due to stress shielding and low heat transfer effects. No such pattern appeared in the depth prediction errors from the same test set (Figure 4e see Figure S4, Supporting Information for equivalent temperature error distributions). The error distribution was homogeneous, with very few localization errors (just 4.2%) exceeding 4.4 mm. The separation of adjacent grid lines suggested a better localization than it was possible to achieve by looking at the raw response magnitudes in Section 2.1. This figure rises to 28.2% for the large sensor: though only 3.6% of errors exceed the strain sensor separation. To illustrate this, Figure 4f demonstrates the accuracy of the small skin sensor's network when a circle of random temperature/depth and 20 mm diameter is transcribed. The locations were accurately identified, and the reconstructed circle was clear, with more noise in the lighter depths. Such localization would be not possible without the biomimetic network.

**Figure 5:**
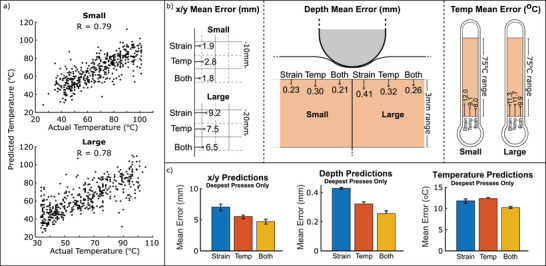
a) Predicted versus actual temperatures for all presses in the test sets (*n* = 500) of the small and large skins. The correlation coefficient, *R*, is calculated for each, which demonstrates a clear positive correlation. b) Training the neural networks using only responses from the strain sensors, only responses from the temperature sensors, or a combination of both. For all predictions and skin sizes, using both inputs produces the lowest errors. c) Training the large skin's neural networks using only the deepest presses (2.5–4 mm).

**Figure** **5**a investigates the success of temperature predictions by plotting the predicted versus actual temperatures for the small and large skin. Looking at the raw data response, it was expected that based on heat transfer limitations, the small skin should achieve more accurate predictions. However, the accuracy difference was not significant for the two sizes. The positive correlation coefficients of 0.79 and 0.78 for the small and large sizes, respectively. The similarity of the two temperature sensing performances was also observed in Figure 4b. In this case, it was seen that the large skin had slightly more outliers than the small skin, which marginally increased the mean error. Since the biomimetic design of the skin placed the temperature sensors on the surface, heat could transfer quickly to the temperature sensors during the pressing period for both skin sizes. To further improve the accuracy for the two skin sizes, longer presses, or a more thermally conductive substrate, could minimize this effect by improving the transmission of temperature information across the upper layers.

The improvement in a network's predictions compared to Section 2.1's naive estimations is due to its ability to simultaneously analyze and identify patterns in large quantities of data. It was seen in Figures 2 and 3 that the strain and temperature responses of the skin were not independent, and both sensor types were affected by changes in both strain and temperature. It was expected that the networks with a combination of both types of sensor responses would yield the most accurate predictions. This effect is demonstrated in Figure 5b, which shows the test set's mean error for localization, depth, and temperature predictions of the trained network. Each bar shows the average and range of errors for a different network, trained with the data from all sensors, only strain sensors or only temperature sensors. In all six cases, the redundancy provided by using both sensor types at the networks' input gave the lowest error in the bimodal system. For the small skin, the behavior of the individual sensory inputs was as expected. The strain input yields a lower mean error than the temperature input for the localization, and the temperature input yields a lower mean error for the temperature prediction than the strain input.The small skin clearly showed the benefits of coupling the two sensory inputs. Whilst pure strain responses can be used to train better localization/depth networks than temperature responses, the combination of the two gives the best response. Similarly, temperature errors were lower on a network trained with temperature responses over one trained with only strain responses, but the combination had higher accuracy.

For the large skin, the results were not as expected. While using both inputs combined, yielded the expected outcome of having the smallest error, looking at the individual responses for each input the error values were not anticipated. The temperature inputs alone gave lower test set errors than the strain inputs for localization and depth predictions. At the same time, the strain input yielded a lower mean error for the temperature prediction than the temperature inputs. To understand better these contradicting results, an additional test was performed for the large skin. From the testing of the individual points, it was seen that the uppermost temperature layers proved useful in detecting and localizing the light touches. For that reason, the same investigation was repeated to include only the data points from the higher valued of depth (larger than 2.5 mm). This alteration resulted in a reduction of the mean error for the x/y localization, but for the other predictions the results didn't change (Figure 5c). Thus, it was concluded that the large interelement distance resulted in areas in the skin with high values of error that led to a larger value of the mean error, especially for temperature sensing. Therefore, it is evident that the interelement distance is crucial for achieving accurate predictions. Even though this observation indicated a strong presence of sensor interdependence that was crucial for improving predictions in the small skin, the bimodal sensor cross‐talk yielded the best prediction accuracy for both sizes. Overall, combining the two different sensory types has the significant benefit of reducing the prediction error compared to using only one sensory input, regardless of the interelement distance.

## 3 Conclusion

In this work, the performance of a multimodal biomimetic skin capable of detecting and quantify the locations, depths, and temperatures of tactile stimuli was demonstrated. The sensory structure of human skin was used as a design guide.

Same as natural signals are transmitted to the somatosensory cortex, neural network processing provides adaptive sensing, capable of learning the non‐linearities of mechano‐ and thermoreceptor signals and localizing stimuli to sub interelement resolutions. The depth of the sensing elements significantly affected the localization of the stimulus. Additionally, the magnitude of the stimulus was dependent on the position and depth of the sensing elements on the skin and particularly for the top layers, the response was not selective to each stimulus. The sensor response was selective when the sensors were used as a single sensing element. When the sensors were integrated in a substrate and used over an area, there were significant limitations in the localization of the stimulus, particularly in the substrate area between neighboring elements. In this area, temperature recognition was not possible for large interelement distances. The neural network processing enabled the stimulus recognition and localization over the entire area of the skin, regardless of the proximity to the element. Despite the algorithm allowing stimulus recognition in areas that were not possible to detect before, the prediction accuracy showed a dependency on the interelement distance. The uppermost thermoresistive elements, which reflect the free nerve endings found in the skin's epidermis, provided not only thermal feedback from tactile stimuli, but also valuable information on the location of lighter touches that may minimally deform the lower mechanoreceptive elements. The lowermost mechanoreceptive elements represent the functionality provided by the Pacinian corpuscles and Ruffini endings for the detection of deep pressure and stretching. In combination with the upper elements, the light touch localizations of Merkel's discs and Meissner's corpuscles become reproducible with the help of neural network processing. It was also seen how multi‐modal sensor fusion through the network architecture led to higher accuracy when there was a cross‐talk between the thermoreceptive and mechanoreceptive sensing elements. In addition, this tunable architecture suggests the potential to adapt to dynamically changing environments in further implementations. Future work will aim to process the signals to similarly represent the full functionality that these provide in nature, including the recognition of high and low frequency vibrations and the separation of cutaneous stretching from associated signals. This combination of these behaviors into truly multi‐modal skin would significantly improve the potential and capabilities of both sensorized wearables and prosthetic devices.

## 4 Experimental Section

### Processing of the Thermoreceptive and Mechanoreceptive Sensing Elements

Carbon black (Ensaco 260G) obtained by Imerys (Paris, France) was mixed with a thermoplastic elastomer materials like Styrenic thermoplastic elastomer (TPS) and thermoplastic polyurethane (TPU). TPS with a shore hardness of 50A, obtained by Kraiburg TPE GmbH and Co (Waldkraiburg, Germany), was used as a matrix material for the mechanoreceptive sensing elements. A TPU with a shore hardness of 85A (BASF, Ludwingshafen am Rhein, Germany) was used for the thermoreceptive elements. The mechanoreceptive composite was prepared by mixing TPS and carbon black in 45% w/w concentration with a torque rheometer from Thermofisher (Karlsruhe, Germany) at 190°C. The thermoreceptive element composite was prepared by mixing TPU and carbon black in 20% w/w concentration with a torque rheometer from Thermofisher. The carbon content for both sensors was selected based on the findings from an older study.^[^ ^58^ ^]^ The resulted composites were extruded to a filament with a capillary rheometer from Netzsch Gerätebau (Selb, Germany) in the form of 1.75 mm filaments and then cut into pellets.

### Fabrication of the Sensorized Skin With Pellet‐Based Material Extrusion

For the fabrication of the sensorized skins with integrated thermoreceptive and mechanoreceptive elements, thermoplastic material extrusion based additive manufacturing (MEX‐AM) was used. This is the new name according to ASTM ISO/ASTM 52900, which should replace the name fused deposition modeling (FDM) and fused filament fabrication (FFF) in the future. A pellet‐based extruder Voladora NX+ pellet printer (International Technology 3D Printers, S.L., Spain) was used to print the conductive TPE‐comoposite materials. Details about the processing had been described in details previously.^[^ ^60^, ^61^ ^]^ For the substrate material, a thermoplastic polyurethane (TPU) with Shore hardness 70A (BASF, Ludwigshafen am Rhein, Germany) was used. The sensing elements were printed in parallel lines on top of the soft substrate. Every line consists of two layers. The width of the sensing elements is 1.6 mm. The elements on L1 were identified as 0t,1t,2t,3t; the elements on L2 were identified as 4t,5t,6t,7t; the elements on L3 were identified as 0s,1s,2s,3s, the elements on L4 were identified as 4s,5s,6s,7s. Two different sizes were produced (4 cm, 8 cm) for the skins with a square geometry. The interelement distance, i.e., the distance between two neighboring sensing elements in the same layer, was 20 mm for the large skin and 10 mm for the small skin. The four layers in total were fused together with a SilPoxy by Smooth‐On (Macungie, Pennsylvania, USA) adhesive. Two layers with mechanoreceptive sensors were positioned at the bottom and two layers with thermoreceptive sensors on top. The sensing elements were connected using a silver yarn. The connections with the silver yarn were made using the Bare Conductive Paste from RS Components (Corby, UK).

### Sensor Characterization

A Universal Robots UR5 robotic arm was used for the characterization of the sensor performance. Its silver steel probe (5 mm diameter, hemispherical tip) had an integrated heating device that could heat up the tip of the probe in a controllable manner in the range (30, 100)°C. An internal 10 kΩ thermistor (RS Pro, Corby, UK) was used to record the ground truth temperature values throughout the experiments. A characterization area was defined within the skin to ensure that the surface could be deeply probed without damaging the peripheral connections. The e‐skin was adhered to an elastomeric substrate made with the silicone rubber EcoFlex 00‐30 with 2.4 mm height. An acrylic frame (Figure S1, Supporting Information) was used for stabilizing the substrate and the skins during the probing with the robot arm (Figure S5, Supporting Information), to which the skin was attached using tape. One end of every sensor line was supplied with +5V (DC) and a fixed resistor, and the other with the ground of a potential divider. The voltages at each of the 16 central nodes were recorded using a data acquisition module USB‐6212 from National Instruments (Austin, Texas, USA) with a measurement frequency of 20 Hz.

Two types of measurements were performed:

1. *Defined point measurements*. Three points (A, B & C in Figure 2a, scaled correspondingly to each grid size) were chosen, and probed at a depth of 1 mm or 4 mm, and a temperature of 50 °C or 100 °C. All tests were repeated 5 times. Each press was held for 10 s, with 6 s pauses between repetitions. Point A was selected as the intersection between two temperature sensing elements (6t and 2t). Point B was selected as the intersection between two mechanoreceptive sensing elements (2s and 7s). Point C was selected as the intersection between a thermoreceptive and mechanoreceptive sensing element (0t and 4s). For the tests, the robot arm pressed the sensorized skin in the defined locations for 10s and then released it.
2. *Random point measurements*. When collecting data for training the neural networks (see Section 4), x/y coordinates were randomized within the characterization area, and a random depth between 1 and 4 mm was selected. 4500 measurements were taken in succession, using the same timings as the defined point measurements. To avoid transient effects caused by randomizing the probe temperature each time, the probe temperature was oscillated between 30 and 100 °C throughout the collection.

### Neural Network Implementation

Feedforward neural networks were used for predicting the location, depth, and temperature of the tactile stimulus. For each probe cycle, the ground truth values were saved for the temperatures, x, y, and z coordinates. The responses of the 16 sensor lines were simultaneously recorded and used as inputs to the network. As the sensors suffer from temporal non‐linearities like hysteresis and drift, past data points from each of the 16 sensor lines were also provided to the network for prediction. This can also be achieved by using recurrent neural networks which have an internal memory system. Several preliminary tests were performed to compare different architecture types (Figures S6 and S7, Supporting Information). Finally, an input layer size of 96 was selected. This corresponds to six inputs per sensor response, which were directly sampled from the sequence of measured voltages: two values just before pressing, two values whilst pressed, and two values just after pressing (Figures S2– S4, Supporting Information). Three hidden layers were used, of sizes 200, 50, and 20, each using a tanh activation function. A final regression layer of size four predicted the x/y coordinates, depth, and temperature of the press.

4500 data points were used for training the neural network, split into 80% training, 10% validation, and 10% test sets. Before training, each input was normalized to have zero mean and unity standard deviation, and each output was scaled to fall in the range (0, 1). These same normalization parameters were applied to all subsequent sensor responses fed to the networks. Training used stochastic gradient descent with momentum, and mini‐batches of size 500. An initial learning rate of 0.2, dropping with a factor of 0.05 every 500 epochs, was used until the validation loss (calculated every 30 iterations) had not decreased over 100 consecutive calculations. The training was done using MATLAB's Deep Learning Toolbox.

### Statistical Analysis

For all the data analysed in this paper, no pre‐processing of the data was done. General trends were shown as mean ± SD, unless specified otherwise. Sample size for each statistical analysis was provided with each figure. Statistical tests to quantify significance of results were not done. All data analysis and plotting was done using MATLAB programming environment.

## Conflict of Interest

The authors declare no conflict of interest.

## Supporting information

Supporting Information

## Data Availability

The data that support the findings of this study are available from the corresponding author upon reasonable request.
