# Supplementary material for: Sensorized Skin With Biomimetic Tactility Features Based on Artificial Cross‐Talk of Bimodal Resistive Sensory Inputs
Source: Adv Sci (Weinh). 2023 Sep 7;10(30):2301590. doi: 10.1002/advs.202301590 (PMC10602557; doi:10.1002/advs.202301590)
Supplement: Supplementary file 1 — Supporting Information [file ADVS-10-2301590-s001.pdf]

ADVANCED SCIENCE
Open Access

Supporting Information

for *Adv. Sci.*, DOI 10.1002/advs.202301590

Sensorized Skin With Biomimetic Tactility Features Based on Artificial Cross-Talk of Bimodal Resistive Sensory Inputs

Antonia Georgopoulou, David Hardman, Thomas George Thuruthel, Fumiya Iida and Frank Clemens**

Sensorized skin with biomimetic tactility features based on artificial crosstalk of bimodal resistive sensory inputs: Supplementary Material

S1 Supplementary Figures

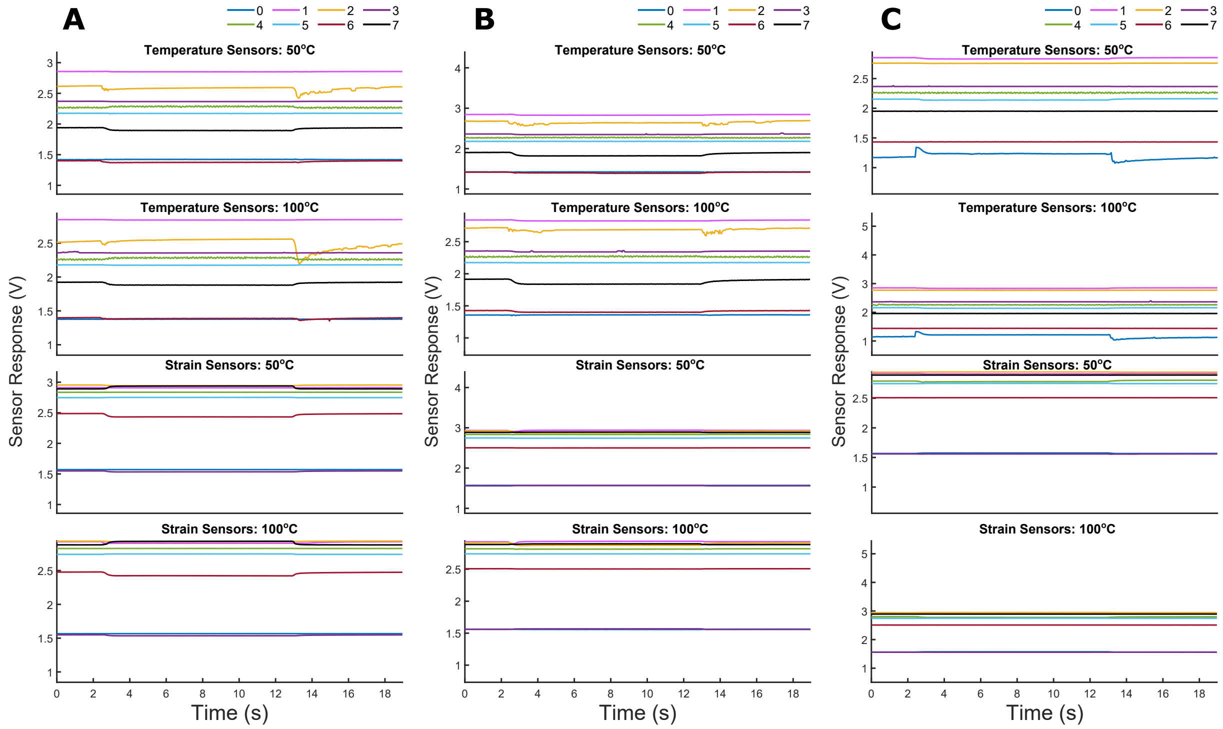

Figure S1: The time-series responses of the channels to deep presses, used to generate Figures 2c & d.

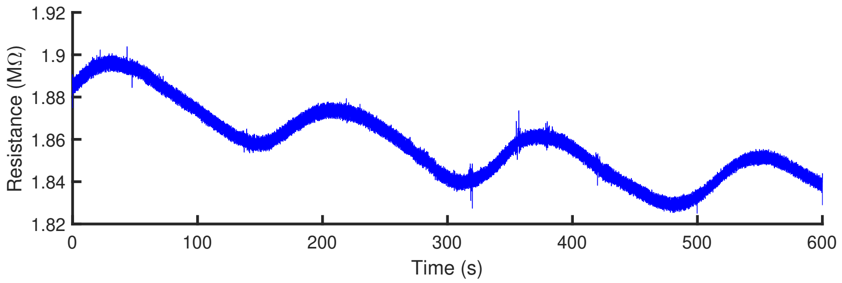

Figure S2: The resistance of temperature sensor 2t measured over a 10 minute period, during which a 4 mm press at position A is held and the probe's temperature varied sinusoidally between 30 °C and 100 °C.

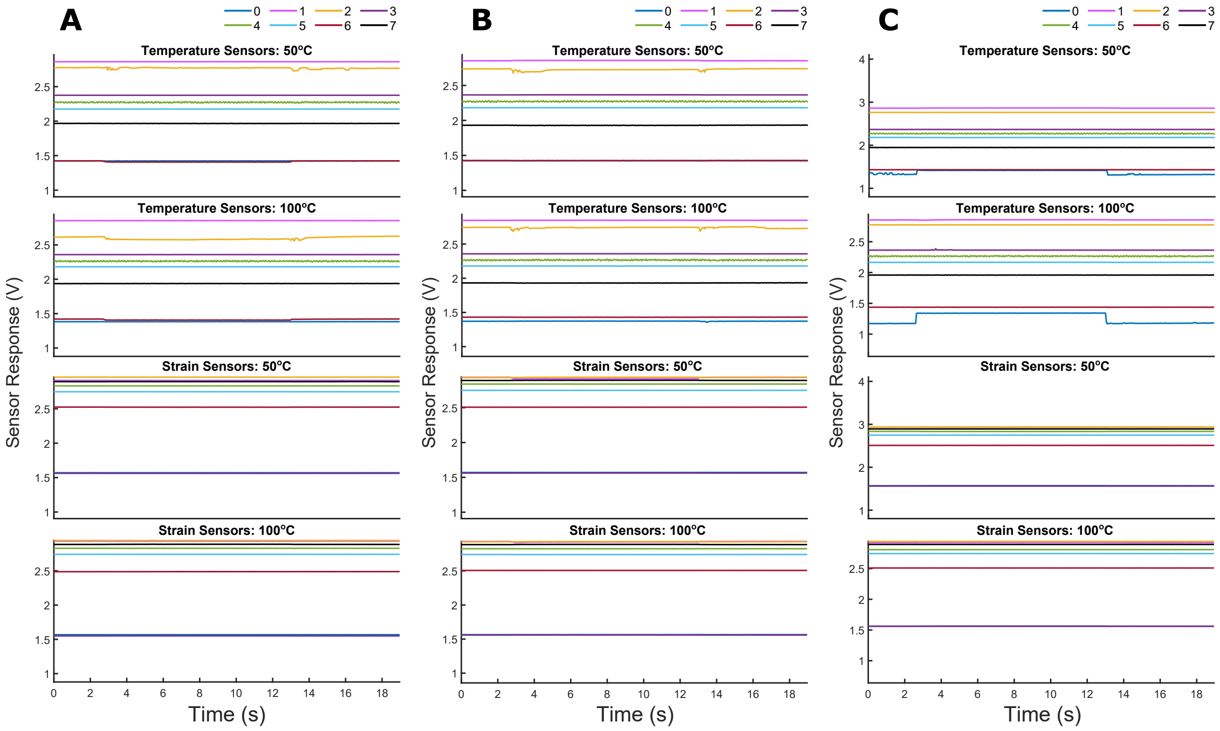

Figure S3: The time-series responses of the channels to light presses, used to generate Figures 2e & f.

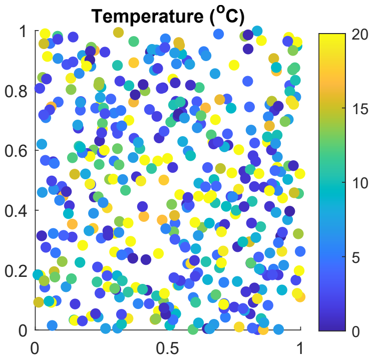

(a) Small

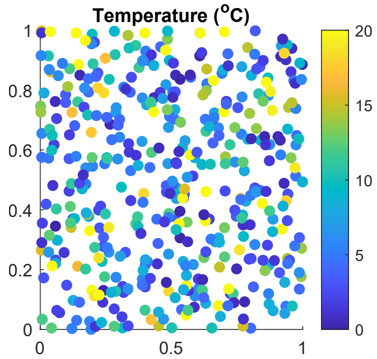

(b) Large

Figure S4: Temperature error distributions for the networks trained on the small and large skins. Note that the cut-off value is set to 20°C to best visualize the distribution, though a number of errors exceed this.

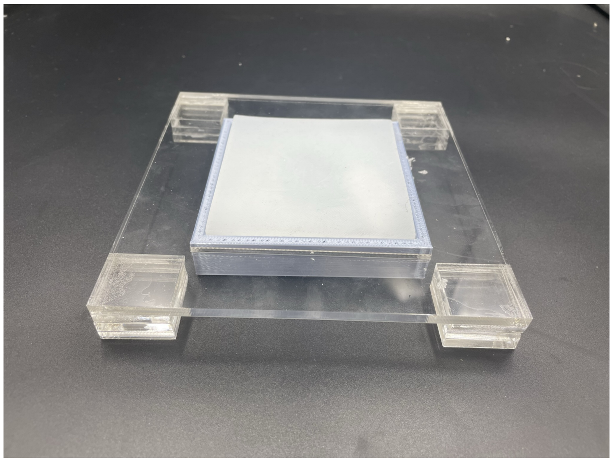

Figure S5: Underlying PMMA support frame & substrate

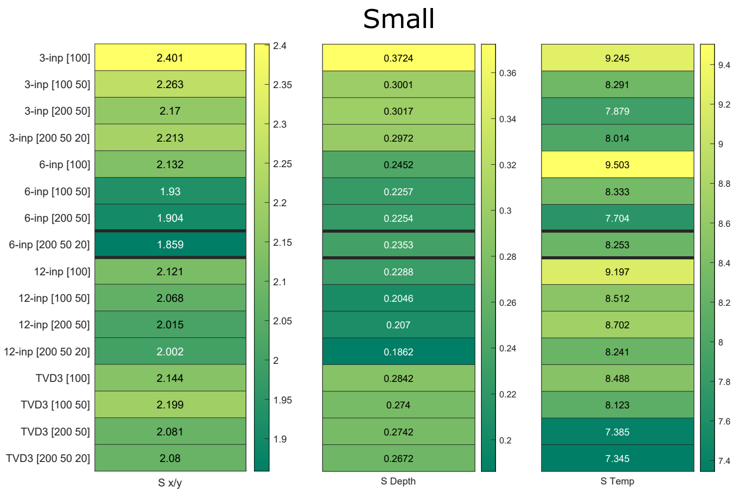

Figure S6: Small architecture tests: average test set errors. Four different hidden layer architectures are tested, with sizes [100], [100 50], [200 50], & [200 50 20]. Different sizes of response data input are also tested: 3 samples (with and without total variation denoising), 6 samples, and 12 samples. The 6-input [200 50 20] architecture is chosen for use in experiments.

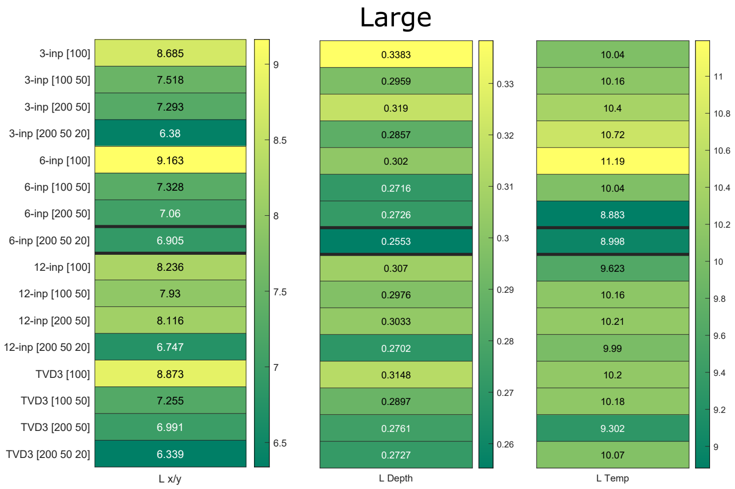

Figure S7: Large architecture tests: average test set errors. Four different hidden layer architectures are tested, with sizes [100], [100 50], [200 50], & [200 50 20]. Different sizes of response data input are also tested: 3 samples (with and without total variation denoising), 6 samples, and 12 samples. The 6-input [200 50 20] architecture is chosen for use in experiments.

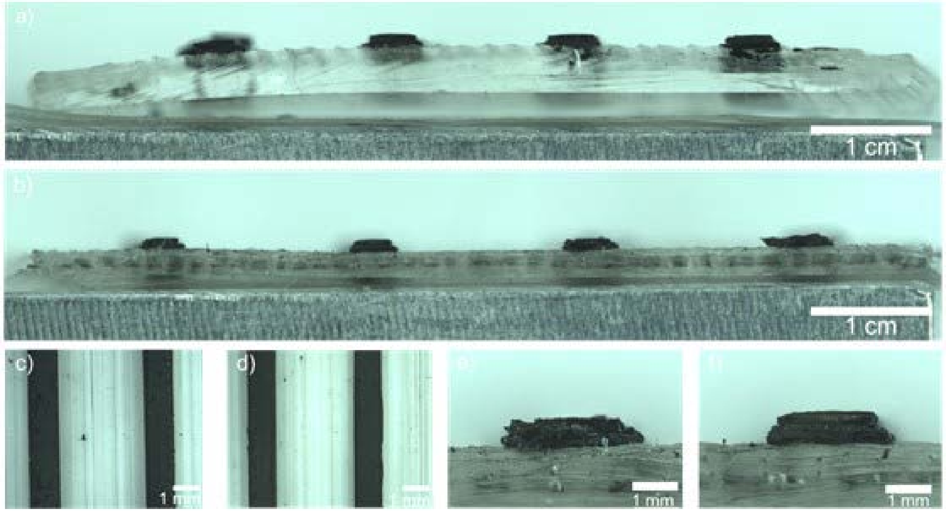

Figure S8: Microscope pictures of a)-c)-e) thermoreceptive and b)-d)-f) mechanoreceptive sensing layers. Pictures obtains with the microscope Zeiss Stereo Discovery (Carl Zeiss Microscopy, Jena, Germany).
